# Design, Manufacturing and Properties of Refractory Materials

**DOI:** 10.3390/ma17071673

**Published:** 2024-04-05

**Authors:** Ilona Jastrzębska, Jacek Szczerba

**Affiliations:** Faculty of Materials Science and Ceramics, AGH University of Krakow, al. A. Mickiewicza 30, 30-059 Krakow, Poland

With pleasure, we present this Special Issue of *Materials*, titled “Design, Manufacturing and Properties of Refractory Materials”. Refractory materials are both economically and socially strategic materials as they enable the production of other crucial products, including steel, non-ferrous metals, cement clinker, lime, glass and many others. They are designed to operate at high temperatures, temperature gradients and under severe chemical and mechanical loadings. Therefore, extensive research of their properties is critical in the development of new types of longer-lifetime and pro-ecologic materials as well as in enhancing the properties of existing ones. At present, artificial intelligence contributes progressively to making refractory production and application more sustainable, which is one of the scopes of this issue. This Special Issue consists of 18 papers, including 1 review article on the application of machine learning in the refractories field, which is the first review work on this modern topic so far. This issue covers the following research aspects in refractories: artificial intelligence and computer-aided methods, simulation of refractories properties, new refractory materials, castables and binders, corrosion, and, finally, it is closed with environmental aspects in refractories. We hope this issue will bring new insights into the development of refractories, allowing them to serve us better and longer.

## 1. Artificial Intelligence and Computer-Aided Methods

Sado et al. [1] presented a review article on the application of machine learning (ML) technology in the investigation of MgO–C refractories from the perspective of their key properties prediction. Their work also presented different ML algorithms currently used in materials engineering. The authors critically assessed works on the up-to-date application of ML in the refractories field, which is currently developing rapidly. Challenges in the development of reliable models were discussed.

Zelik et al. [2] used Bayesian modeling for the prediction of MgO–C refractory unit wear rate in the slag-spout zone of a 350 t-oxygen converter that completed 2063 heats, applying data of lining thickness collected via laser measurements. A 420 µm usage of refractory thickness per one heat of convertor was determined using this type of modeling.

By using neural networks—an artificial intelligence tool—Janĉar et al. [3] ordered parameters which positively and negatively impact the refractory lining in a steel ladle. He showed that the time of empty ladle most adversely affects the a ladle’ campaign due to thermal shocks of refractory lining. Other parameters which were observed to shorten the lifetime of the lining are electrical energy consumption, number of vacuum heats and temperature of steel tapped to the ladle. On the other hand, Ar consumption and time of full ladle were found to positively influence the lifetime of refractory lining.

Stec et al. [4] used the innovative technique of corrosion testing combining hot metal penetration with subsequent X-ray computed tomography to investigate the microporous carbon refractory lining dedicated to blast furnace hearth walls. The results showed the volume percentage of permeable pores and metal inclusions of 2.5% and 3.2%, respectively, with their respective maximum sizes of 410 µm and 843 µm. This shows that during 1 h-penetration at 1500°C crude iron dissolved the carbon skeleton of the refractory lining.

Jastrzębska and Piwowarczyk [5] showed how computer image analysis can be utilized to extract more valuable data from SEM images via traditional stereology-based methods vs. automated method developed in the work for spinel refractory material. The automated image analysis allows for fast retrieval of the data, like quantity of phases at SEM image, which in some cases—like partly amorphous materials—is complementary to the XRD method.

## 2. Simulation of Refractory Properties

Grigoriev et al. [6] presented computer modeling of 96% SiO_2_ refractories (for coke ovens, heat exchangers of blast furnaces, and glass-making furnaces) using the method of homogeneously deformable discrete elements to simulate the uniaxial compressive and tensile failure in a wide range of quasi-static and dynamic loading rates. Also, crack patterns were presented for real and model materials. They determined the real and virtual contribution of cracks within grains, matrix and along the interface. This is important as brittleness at failure correlates with an increase in the relative crack length along the grain/matrix interface. For composites, which are refractory materials, the strength of the interface between the matrix and reinforcing constituents (large grains, fibers) strongly correlates with the material’s strength, but an extremely strong interface leads to brittle failure.

## 3. New Refractory Materials

Two articles presented by Zienert et al. [7] and Storti et al. [8] show a novel Nb-Al_2_O_3_ composite aggregate which can be used for the production of refractories with enhanced shock resistance. Cold crushing strength measured at 1300 °C revealed their semi-plastic behavior. Also, these novel composites can be candidates for new high-temperature heating elements. This area seems to have plenty of room for further research.

## 4. Castables and Binders

αAl_2_O_3_ hollow spheres with sizes 5–100 µm were introduced by Stonys et al. [9] to bauxite refractory castables to check their influence on castable thermal shock resistance. In their study, additions of 2.5% and 5% of hollow Al_2_O_3_ were found to improve the thermal shock resistance of castables, which withstood 30 thermal cycles, whereas a 10% addition caused their deterioration due to clustering, poor bonding of spheres with the surrounding matrix and formed irregular-shape voids. Thermal shock resistance was tested by heating them at 950 °C and water quenching, and this was assessed by determining their loss in ultrasonic pulse velocity.

Tang et al. [10] investigated the influence of CaTiO_3_, MgTiO_3_ and nano-TiO_2_ additions on the physical and mechanical properties of alumina-magnesia castables in order to increase castable densification, thus, inhibiting the effects of CA_6_ and spinel expansion. They found that MgTiO_3_ is unstable in all tested materials, while 2 wt.% CaTiO_3_ results in the best castable properties. The latter one, after being subjected to 1450 °C, received linear shrinkage of −0.5%, apparent porosity of 12%, bulk density of 3.2 g/cm^3^, CMOR of 45 MPa, and Young’s modulus of 194 GPa, which is significantly better in comparison to the castables with nano-TiO_2_ and MgTiO_3_ additions.

Zemanek and Nevřinová [11] tested the performance of castables with the use of silica sol and compared them to castables with calcium aluminate cement. They found that sol-bonded castables perform successfully compared to traditional calcium aluminate-bonded castables in terms of permanent linear change, compactness and corrosion resistance.

Wang et al. [12] tested the influence of 5 µm-andalusite addition on thermal shock resistance of Al_2_O_3_–SiC–C castables dedicated for the runners of blast furnaces. They found noticeably improved oxidation resistance and residual CMOR after 10 thermal cycles compared to castables without the addition. Such improvement was related to an enhanced mullitization of andalusite, which started at 1250 °C and finished at 1450 °C. Thus, the formation of a liquid phase (due to andalusite decomposition) was enabled earlier, which densified the microstructure via the crystallization of secondary mullite. The maximum 5% addition of micro-andalusite resulted in over 40% higher residual CMOR after 10 thermal cycles at 950 °C and water quenching, and 2% lower open porosity (20%) when compared to the reference. With respect to the previous work of the authors, the oxidation resistance of castables with a 5% addition of micro-andalusite was over 30% better than a 19% addition of andalusite 1–3 mm.

Inorganic chemical binders for refractory materials were characterized by Hopp et al. [13] from the perspective of structure–property relations. The presented work excellently shows the comparative instrumental methods that can be applied for the complete characterization of binder action mechanisms, including XRD, FTIR, Raman spectroscopy, NMR and DMA (dynamic mechanical analysis). In particular, phosphate and silicate binders were deeply analyzed.

## 5. Corrosion

Ovćaćíková et al. [14] investigated alkali corrosion, which commonly accompanies aluminosilicate refractories working in boilers for wood biomass combustion. The ashes generated during such biomass combustion are rich in SiO_2_, CaO, Al_2_O_3_, Fe_2_O_3_ and alkalis like Na_2_O and K_2_O. Moreover, they tend to slag, thus, forming a sticky layer of ash particles on the surface of the refractory lining. After a corrosion test at 1200 °C for 2 h, SiO_2_–Al_2_O_3_ refractories with a SiC addition were found to better protect the refractory lining from the alkali corrosion process compared to traditional aluminosilicate refractories.

Darban et al. [15] revealed the corrosion of an alumina-spinel refractory against secondary steel slag via a coating corrosion test at 1350 °C and 1450 °C. They summarized the study by showing the passive corrosion mechanism of the refractory, with the formation of CA, CA_2_ and CA_6_ layers around the Al_2_O_3_ core and C_2_AS in the matrix.

Ludwig et al. [16] investigated the corrosion of Cr_2_O_3_-bearing refractory raw materials against PbO-rich copper slags via hot-stage microscopy. They found that among the four tested raw materials—namely, two magnesia-chromite co-clinkers, Pakistani chrome ore and fused spinel—the latter was characterized by the lowest chemical resistance expressed by the lowest melting and highest final shrinkage (8%). Forsterite, Mg_2_SiO_4_, was the main corrosion product for all the tested materials.

## 6. Environmental Aspects in Refractories

Interesting from the environmental point of view are the results shown by Xu et al. [17], who investigated alumina castables bonded with calcium aluminate cement, in which Cr was introduced to composition in the form of pre-synthesized solid solution (Al_1-x_Cr_x_)_2_O_3_. Chromium (III) — previously bounded in this solid solution—is therefore much less prone to oxidize and form Cr(VI) compounds, and this effect is especially enhanced when more CA_6_ is present in the system. This smart approach can greatly help to reduce the negative impact of the utilization of Cr_2_O_3_ in refractory materials.

Nguyen and Sokolář [18] presented the concept of utilizing fly ash as a source of Al_2_O_3_ to create in situ spinel in new forsterite refractories. The formation of spinel helps to decrease the global, large expansion of forsterite-based materials. They revealed that refractoriness under load was at least 1500 °C (0.5% deformation), HMOR above 20 MPa, and corrosion resistance (expressed as penetration depth) was up to 2 mm for Fe, 3 mm for the clinker, 1 mm for Al and 0.01 mm for Cu. Thus, these materials show the perspective to be adapted in the Cu industry. Therefore, a further investigation of coarse-grain material is needed.
